# Reply to: “Questions remain about the biolability of dissolved black carbon along the combustion continuum”

**DOI:** 10.1038/s41467-021-24478-x

**Published:** 2021-07-13

**Authors:** Yuanzhi Qi, Wenjing Fu, Jiwei Tian, Chunle Luo, Sen Shan, Shuwen Sun, Peng Ren, Hongmei Zhang, Jiwen Liu, Xiaohua Zhang, Xuchen Wang

**Affiliations:** 1 Key Laboratory of Marine Chemistry Theory and Technology, Ministry of Education/ Center for Frontier Science of Deep Ocean and Earth System, Ocean University of China, Qingdao, China; 2 Key Laboratory of Physical Oceanography, Ocean University of China, Qingdao, China; 3 Center for Isotope Geochemistry and Geochronology, Qingdao National Laboratory for Marine Science and Technology, Qingdao, China; 4 Key Laboratory of Marine Genetics and Breeding, College of Marine Life Sciences, Ocean University of China, Qingdao, China

**Keywords:** Carbon cycle, Marine chemistry

**Replying to** S. Wagner et al. *Nature Communications* 10.1038/s41467-021-24477-y (2021)

Black carbon (BC) is broadly defined as the product of incomplete biomass and fossil fuel combustion, including biochar and soot^1^. While this definition is intuitively clear, the exact chemical composition and structure of this ubiquitous material are difficult to differentiate. With many proposed pathways of BC formation, it is now well accepted that BC is carbon-rich, structurally condensed, and contains abundant aromatic compounds such as polycyclic aromatic hydrocarbons (PAHs). The terminology of BC has also been confused with pyrogenic carbon (PyC) and elemental carbon (EC). We feel that PyC is probably the best description of this material that eliminates the confusion based on its chemical composition that has never been precisely determined.

Dissolved black carbon (DBC) is the fraction of BC dissolved in natural aquatic systems and has been studied extensively in recent years due to its potential importance in global carbon cycling. However, isolation of DBC in natural waters and determination of its chemical composition is more challenging^2^. The literature to date has been exclusively based on the methods used for the isolation and quantification of DBC which has led to inevitable uncertainty and controversy^2,3^.

Using the solid-phase extraction (SPE) and chemothermal oxidation (CTO) method^3^, we investigated DBC in four large and two small mountainous rivers in China; the Yangtze River and Yellow River estuaries; the East China Sea (ECS); and the North Pacific Ocean basin (NP). We measured carbon isotopic (^13^C and ^14^C) values of DBC and found that the carbon isotopic signatures of DBC are relatively homogeneous. Further, DBC ^14^C in rivers is predominantly young derived mainly from biochar, aging during continuum transport, and cycling in the ocean^4^. Combined with results from biochar leaching and degradation experiments, we found that DBC is dissolved from biochar and degraded by bacteria in river water. This suggests that a fraction of riverine DBC could be labile and respired during transport and mixing into the ocean as well as that residual DBC is likely cycled and aged on the same time scales as bulk dissolved organic carbon (DOC) in the ocean^4^. After the publication of our study, a Matters arising (MA) article^5^ raised concerns regarding the bio-liability of DBC as we proposed. We respect these concerns and the following is our response.

We feel that the main controversy lies in the uncertainty of the methodology and the one-sidedness of the definition of DBC. First and foremost, we believe that the experiments we conducted were robust and support the conclusion that the removal of the dissolved compounds leached from biochar was due to microbial biodegradation as supported by the bacterial abundance measurements. During the long-term incubation (1700 days), we compared the biochar leachate in bacteria-inhibited river water to the biodegradation of biochar leachate in bacteria-active river water^4^. The degradation is real. Obviously, we could not create duplicates for each treatment but the data from the experiments was not a single-point measurement. It was a long-term experiment with continuous measurements and consistent results. This kind of experiment has been used to examine biochar solubility^6^. Because the well-burned locust wood biochar, by definition, was BC, we believe that compounds leached from the biochar were a fraction of BC but we did not determine whether they were aromatic compounds or not. Previous studies have well demonstrated that both low-molecular-weight (LMW) compounds and aromatic compound (PAHs) were leached from biochar in natural environment^7–9^ and the biodegradation of PAHs was identified a long time ago^10^. The question is should we define the uncharacterized biochar leachate in our experiments as DBC or something else.

A second controversy between our studies is related to the different methods we used to measure BC (or PyC). The BC definition via the CTO method was well defined^11^ and has been implemented for many BC studies in natural samples. As summarized by Hammes et al.^3^, the advantage of the CTO method provided excellent differentiation between the soot and chars and is best suited for quantifying the most condensed forms of BC. In this case, we believe that the DBC determined in our study was condensed forms of DBC in the rivers, estuaries, coastal, and open ocean waters. We also doubt that the DBC determined by the CTO method could be a false positive. There has been a multitude of publications characterizing BC in the natural environment using the CTO method and the consistent and promising results of BC contents and radiocarbon values showed that false positives are unlikely. Of course, it is debatable whether the CTO method is suitable for DBC characterization or not, which needs to be further investigated.

Finally, the question of ^14^C age inconsistency for oceanic DBC measured by the CTO method and the benzenepoly carboxylic acid (BPCA) method raised in MA is again related to the methodology. Our current ability to quantitatively determine and characterize BC is largely method-specific. Different operationally defined methods could generate very different results^3^. We suspect that the large differences in the ^14^C ages measured for DBC in the ocean could also be method-specific. Using the combined SPE/CTO method, we reported the ^14^C ages of DBC were modern to 1500 years old in the large continental rivers, 390–2600 years old in the ECS, and about the same as the age of bulk DOC (~6000 years) in the deep NP^4^. We predict that DBC transported in rivers is derived mainly from biochar and ages during continuum transport and cycling in the ocean. DBC is likely cycled and ages on the same time scale as bulk DOC in the ocean. We now have some very interesting data of BC (PyC) determined in the sediment solid phase and porewaters in large river-influence coastal seas to support this. While using the combined SPE/BPCA method, it was reported that DBC had ^14^C ages of 23,000 ± 3000 years in the deep ocean^12^. This large age gap of DBC in the ocean is not currently well-understood. Using the broad definition, the ^14^C ages of DBC (or PyC) measured by the CTO method should be exclusively for BC regardless of whether it is comprised of aromatic or nonaromatic compounds. In contrast, the ^14^C ages of DBC measured by the BPCA method characterize benzenepoly carboxylic acids that are then converted to DBC. The major assumption of this method is that these aromatic molecules are exclusively produced from BC, not other sources. Whether this assumption and converting factor are valid or not for SPE-extracted marine DOM is unknown because there is no such reference material to represent DBC in the ocean so no methods used to determine DBC in natural waters have been validated^2^. One study reported that up to 25% of the isolated BC fraction in soils using the BPCA method was produced in situ, without fire or charring, suggesting that the method to quantify BC exclusively formed by pyrogenic processes may be biased by a significant non-pyrogenic fraction^13^. More interestingly, a study reported that the ^14^C ages of BC (~26,000 years) in marine sediments measured by the CTO method were similar to the polycyclic aromatic hydrocarbons (PAHs; ~28,900 years) extracted from the same samples^14^. In the ocean, PAHs could be derived from other sources such as petroleum and natural hydrocarbon seeps. These sources could contribute more aromatic compounds than DBC in the ocean. When mixed into the DOM pool, the separation of aromatic compounds from BC and non-BC sources could be extremely challenging. Indeed, the reported old ^14^C ages of DBC in the ocean are comparable to the ^14^C ages of hydrocarbons (~18,300 years) extracted from the bottom water above the hydrate seeps in the Gulf of Mexico^15^. Clearly, the current methods used to study DBC in natural samples need to be developed and validated^2^. In addition, the lack of representative reference materials, inconsistent terminology, and varied interpretations of BC in natural environments currently create obstacles for comparing results in the literature. We agree that there is a need among the DBC community to establish consistency when reporting DBC concentration and DBC-specific isotopic signatures with cross-laboratory comparison using different methods and to identify representative DBC reference material in natural waters for method validation. Without these, DBC in natural waters will remain a debatable issue. As pointed by Hammes et al.^3^: “The current definition of BC is imprecise. The ultimate values of the various BC quantification methods are not how they compare to one another, but whether they provide useful information for the application for which they are used”.

## Data Availability

No datasets were used for this Reply.
